# The Statin Target HMG-Coenzyme a Reductase (Hmgcr) Regulates Sleep Homeostasis in *Drosophila*

**DOI:** 10.3390/ph15010079

**Published:** 2022-01-10

**Authors:** Ahmed M. Alsehli, Sifang Liao, Mohamed H. Al-Sabri, Lukas Vasionis, Archana Purohit, Neha Behare, Laura E. Clemensson, Michael J. Williams, Helgi B. Schiöth

**Affiliations:** 1 Department of Neuroscience, Functional Pharmacology Unit, Uppsala Biomedical Center (BMC), Uppsala University, Husargatan 3, Box 593, 75124 Uppsala, Sweden; ahmedm.alsheli@neuro.uu.se (A.M.A.); sifang.liao@neuro.uu.se (S.L.); mohamed.alsabri@neuro.uu.se (M.H.A.-S.); l.vasionis@gmail.com (L.V.); archana31198@gmail.com (A.P.); nbehare@gmail.com (N.B.); laura.clemensson@neuro.uu.se (L.E.C.); helgi.schioth@neuro.uu.se (H.B.S.); 2 Faculty of Medicine, King Abdulaziz University and Hospital, Al Ehtifalat St., Jeddah 21589, Saudi Arabia; 3 Institute for Translational Medicine and Biotechnology, Sechenov Biomedical Science and Technology Park, Sechenov First Moscow State Medical University, Trubetskay Str. 8, 119991 Moscow, Russia

**Keywords:** statins, hmgcr, insulin, sleep, sleep deprivation, mevalonate pathway, circadian rhythm, DH44, corticotropin releasing factor, *Drosophila*

## Abstract

Statins, HMG Coenzyme A Reductase (HMGCR) inhibitors, are a first-line therapy, used to reduce hypercholesterolemia and the risk for cardiovascular events. While sleep disturbances are recognized as a side-effect of statin treatment, the impact of statins on sleep is under debate. Using Drosophila, we discovered a novel role for Hmgcr in sleep modulation. Loss of pan-neuronal *Hmgcr* expression affects fly sleep behavior, causing a decrease in sleep latency and an increase in sleep episode duration. We localized the *pars intercerebralis* (PI), equivalent to the mammalian hypothalamus, as the region within the fly brain requiring Hmgcr activity for proper sleep maintenance. Lack of *Hmgcr* expression in the PI insulin-producing cells recapitulates the sleep effects of pan-neuronal *Hmgcr* knockdown. Conversely, loss of *Hmgcr* in a different PI subpopulation, the corticotropin releasing factor (CRF) homologue-expressing neurons (DH44 neurons), increases sleep latency and decreases sleep duration. The requirement for Hmgcr activity in different neurons signifies its importance in sleep regulation. Interestingly, loss of *Hmgcr* in the PI does not affect circadian rhythm, suggesting that Hmgcr regulates sleep by pathways distinct from the circadian clock. Taken together, these findings suggest that Hmgcr activity in the PI is essential for proper sleep homeostasis in flies.

## 1. Introduction

Statins are among the most prescribed drugs worldwide due to their ability to decrease the risk for cardiovascular disease, the main global cause of morbidity and mortality [1]. Several clinical reports have noted that statins have been linked to sleep disturbances, including insomnia [2], while others report no impact on sleep [3,4,5]. A systemic meta-analysis suggested that statins have no effect on total sleep duration but reduce the wake time or the number of awakenings [6]. Currently, the ability of statins to influence sleep behavior, as well as the mechanism by which statins may affect sleep, is poorly understood.

Sleep is a physiological state regulated by homeostatic, environmental, and circadian pathways, which are conserved from invertebrates to vertebrates. In mammals, the sleep homeostatic process is the primary regulator of the sleep–wake cycle. Furthermore, there are ~10,000 master pacemaker neurons in the hypothalamus that control circadian rhythm in mammals, particularly the suprachiasmatic nucleus (SCN) [4]. In *Drosophila*, several genes, neuronal circuits, and biological processes have been identified as being involved in the regulation of sleep [7,8,9,10]. These include a group of neurons that project to the dorsal layer of the central complex fan-shaped body (dFB neurons), which are proposed to be one effector component of sleep homeostasis [11,12,13]. These neurons are known to receive inputs from Ellipsoid body R2 neurons, which determine sleep drive [13] and suppress movements that may be induced by sensory cue stimulation [14]. Nevertheless, circadian pacemaker neurons that directly synapse the dFB neurons have not yet been reported. There are approximately 75 pairs of neurons in the *Drosophila* brain that have a similar function to mammalian master pacemaker neurons and are distributed bilaterally in several regions of the fly brain, such as the *pars intercerebralis* (PI), ellipsoid body (EB), and clock neurons [14,15,16]. The primary role of these neurons is to determine and shape the sleep-activity profile. In *Drosophila*, the sleep-activity profile is responsible for defining the morning and evening activity peaks that occur under normal 12-h light:dark conditions. Between these morning and evening activity peaks exists a siesta, which normally takes place around midday. There is also an extended period of consolidated sleep at night [17]. 

3-Hydroxy-3-Methylglutaryl-CoA Reductase (HMGCR, in flies this is denoted as Hmgcr), a key enzyme in the mevalonate pathway, is highly conserved in both humans and flies. In humans, this enzyme is an important target for anti-hypercholesterolemia agents, such as statin drugs. Given the high homology between human and fly Hmgcr, the *Drosophila* model is a powerful tool for understanding the role of Hmgcr in the control of sleep behavior [18]. In the present study, we have employed both genetic and pharmacological methods and determined that Hmgcr is required for proper sleep maintenance in *Drosophila*. This work contributes to explaining the mechanisms underlying the effects of statins on sleep, which is of high interest due to the pleiotropic effects of one of the world’s most prescribed medications. 

## 2. Results

### 2.1. Down-Regulation of Hmgcr within Neurons Promotes Sleep

**Figure 1:**
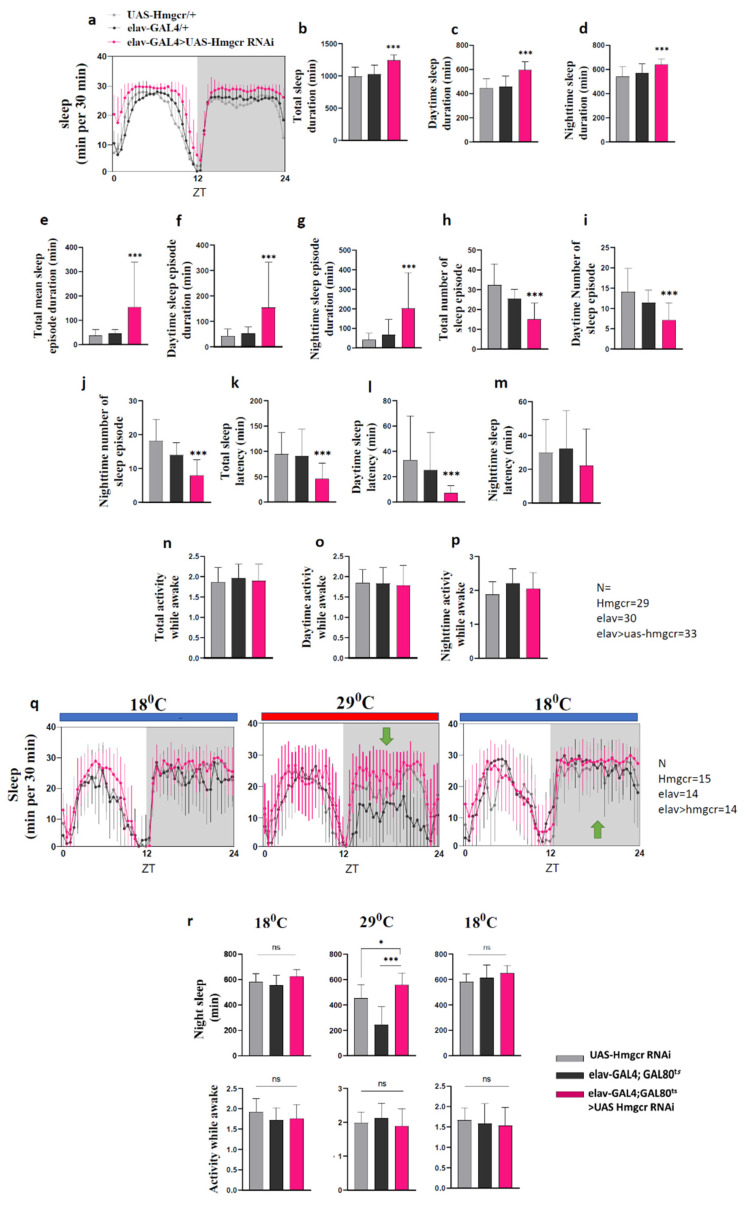
Loss of pan-neuronal Hmgcr in male flies has sleep-promoting effects. (**a**) Sleep diagram per 30 min, (**b**–**q**) total sleep duration, mean and max sleep episode duration, number of sleep episodes, sleep latency, and activity while awake between control groups (*elav-GAL4*\+ and *UAS-Hmgcr* RNAi\+) and the experimental group (*elav-GAL4* > *UAS-Hmgcr* RNAi) have significant differences. All error bars represent ± S.D. For each genotype, *n* = 32 male flies. (**r**) The transgene tubGAL80^ts^ was used to control the expression of *UAS-Hmgcr* RNAi in the *elav-GAL4* neurons. At 18 °C, the GAL80^ts^ blocks the expression of *UAS-Hmgcr* RNAi. At 29 °C, the GAL80^ts^ protein was inactivated, allowing the expression of *UAS-Hmgcr* RNAi driven by the *elav-GAL4*. Transient knockdown of Hmgcr expression by pan-neuronal (*elav-GAL4*) caused a significant increase in night sleep (green arrows). After the GAL80^ts^ protein was reactivated at 18 °C, the increased sleep was reversible and immediately returned to normal (green arrows). All measurements are calculated from the number of beam crossings per minute. In all graphs, error bars indicate ± S.D. For each group *n* = 32 male flies. To detect the significant difference between groups a Shapiro–Wilk test was performed to determine normality, then a one-way ANOVA and Bonferroni post hoc test was performed. * *p* < 0.01, *** *p* < 0.0001.

To determine whether Hmgcr activity is important in the regulation of sleep, the Drosophila pan-neuronal *elav-GAL4* driver [19] was used to knockdown *Hmgcr* expression in fly neurons. The *elav-GAL4* > *UAS-Hmgcr* RNAi flies were less active than control flies (*elav-GAL4* > w^1118^ or w^1118^ > *UAS-Hmgcr* RNAi) (Supplementary Figure S1). This reduced activity was entirely due to an increase in sleep duration (Figure 1a–d), as the activity during the wake time did not differ between *Hmgcr* knockdown and control flies (refer to Figure 1n–p). Further dissection of the sleep behavior showed that loss of *Hmgcr* in all neurons increased the mean sleep episode duration during the day and night but reduced the number of sleep episodes (Figure 1e–g). This led to an overall increase in total sleep duration. Pan-neuronal *Hmgcr* knockdown also reduced total sleep onset latency due to a shortened sleep onset latency during the day (Figure 1q).

**Figure 2:**
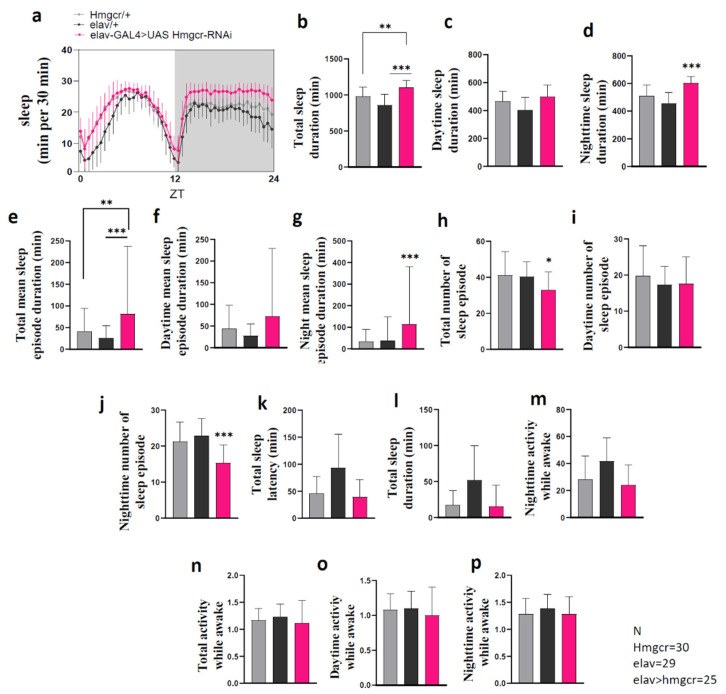
Hmgcr pan-neuronal knockdown in females increases sleep at night. (**a**) Wild-type flies male and females; male flies have a more robust siesta than females. (**b**–**p**) Sleep at night, sleep episode duration, max sleep episode duration, and a number of sleep-episode frequency at night between controls (*elav-GAL4*\+ and *UAS-HMGCR* RNAi\+) and the experimental group (*elav-GAL4* > UAS HMGCR RNAi) have significant differences. Graphs with errors bars indicate ± S.D. *n* = 32 for each group. To detect the significant difference between groups a Shapiro–Wilk test was performed to determine normality, then a One-way ANOVA and Bonferroni post hoc test was performed. * *p* < 0.01, ** *p* < 0.001, *** *p* < 0.0001.

To validate that the apparent sleep-promoting effects were due to a pan-neuronal reduction in *Hmgcr* expression, we performed a second experiment using the *elav-GAL4*, tubulin-GAL80^ts^ transgene to block *UAS-Hmgcr* RNAi expression at low temperature. In addition, we reared the flies at 18 °C to ensure the expression of *UAS-Hmgcr* RNAi was highly suppressed. After shifting the temperature to 29 °C, which inactivated the GAL80^ts^ protein, consolidated sleep significantly increased. When the GAL80^ts^ was reactivated by returning the temperature to 18 °C, sleep was returned to normal levels (Figure 1r). Interestingly, the activity while awake was unchanged. The sleep pattern of female flies is different than males, as females have less daytime sleep compared to males but, similar to males, have consolidated sleep during the night (Figure 2a) [20]. We also found that, similar to male flies, reducing *Hmgcr* pan-neuronal expression in female flies strongly increased night sleep levels (Figure 2b–p). 

**Figure 3:**
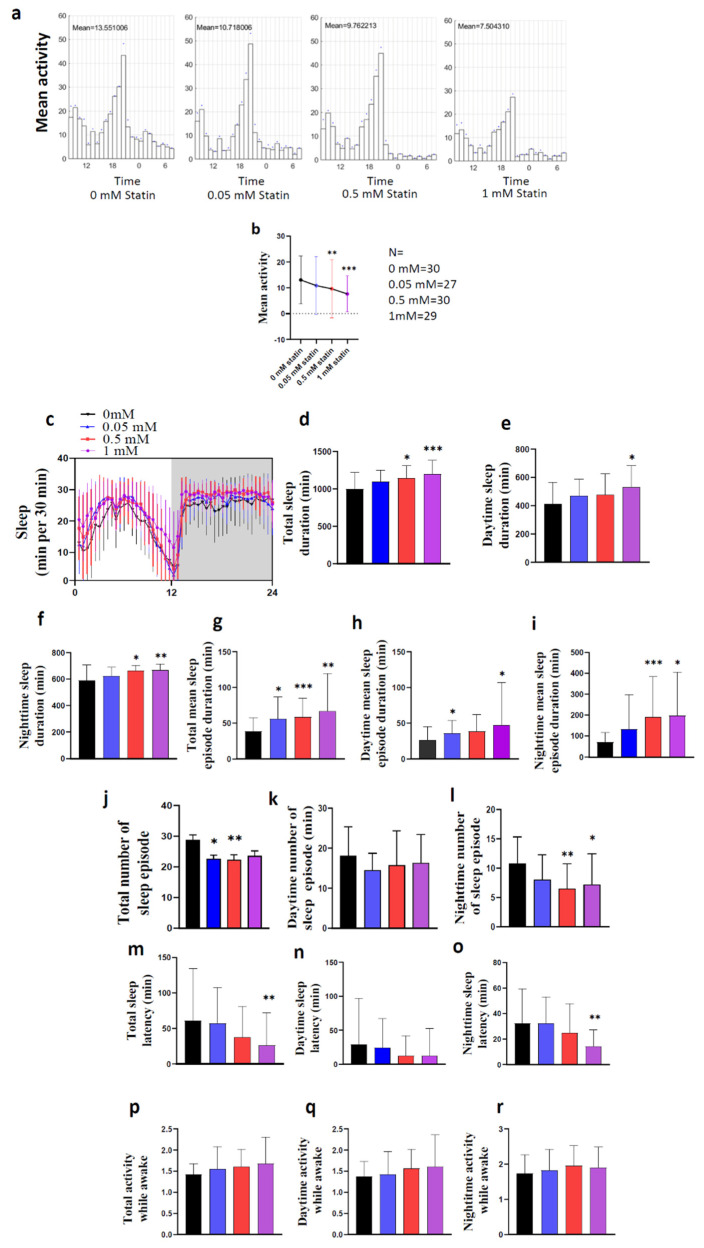
Fluvastatin significantly increases sleep due to increasing episode duration and shortening sleep latency. (**a**–**c**) Activity and sleep data demonstrate the effect of various fluvastatin concentrations on wild-type male flies; this result is for the first day the flies were treated with fluvastatin. The average activity and sleep per 30 min in groups of flies fed with water (black = 0 mM, *n* = 30) or fluvastatin: 0.05 mM (blue, *n* = 27), 0.5 mM (red, *n* = 30), 1 mM (pink, *n* = 29), during 12:12 light and dark periods. (**d**–**r**) Total sleep duration, sleep episode duration, the number of sleep episodes, sleep latency, and activity while awake. All measurements are calculated from the number of beam crossings per minute. The results with error bars indicate mean ± S.D. To detect the significant difference between groups a Shapiro–Wilk test was performed to determine normality, then a one-way ANOVA and Bonferroni post hoc test was performed. * *p* < 0.01, ** *p* < 0.001, *** *p* < 0.0001.

Next, through treatment with fluvastatin, we assessed whether similar effects on sleep behavior would be found after systemic inhibition of Hmgcr activity. Previous publications found that fluvastatin worked well in flies and was able to inhibit Hmgcr activity in the mevalonate pathway [21,22]. Fluvastatin had significant dose-dependent effects (Figure 3a,b and Supplementary Figure S2), where the highest doses increased the amount of sleep duration due to an increase in the mean sleep-episode duration, as well as a decrease in the number of sleep episodes and decreased sleep latency, specifically during the night (Figure 3c–o). Interestingly, locomotor activity during the awake period was unaffected (Figure 3p–r), indicating that the sleep phenotype was not due to low activity. 

### 2.2. Loss of Hmgcr in the Pars Intercerebralis Insulin-Producing Neurons Recapitulates the Pan-Neuronal Phenotype

**Figure 4:**
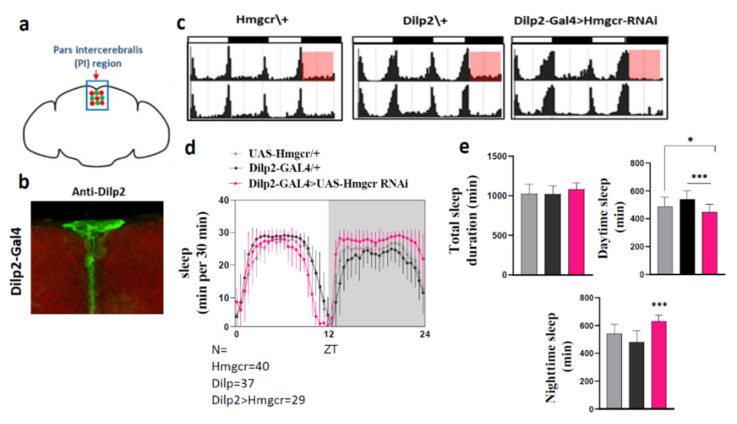
Sleep-activity patterns are affected by Hmgcr knockdown in Dilp2 neurons. (**a**) Diagram of different neuronal cells located in the PI region: these neurons complement each other. DH44 and the insulin-producing cells, green and red circles, respectively. (**b**) PI neurons were stained by anti-Dilp2; the scale bar represents 10 µM. (**c**–**e**) Activity and sleep data for experimental and controls, male flies (*n* = 32) in L:D conditions. (**c**) White bars indicate daytime (ZT0–12); black bars indicate night (ZT 12–24). The genotypes are indicated above the panels. Red boxes denote the locomotor activity during the night. (**d**) Architecture of the sleep parameters. Light area indicates daytime (ZT0–12); gray area indicates night (ZT 12–24). The genotypes are indicated above the graph. (**e**) Graphs indicate locomotor activity, as well as the amount of day and night sleep in minutes. Gray bar = *w^1118^* > *UAS-Hmgcr* RNAi control, Black bar = Dilp2-GAL4 > *w^1118^* control, Pink bar = Dilp2-GAL4 > *UAS-Hmgcr* RNAi experimental. Results with error bars are mean ± S.D. To detect the significant difference between groups a Shapiro–Wilk test was performed to determine normality, then a One-way ANOVA and Bonferroni post hoc test was performed to detect significance for total sleep, daytime sleep, and night sleep. * *p* < 0.01, *** *p* < 0.0001.

Hmgcr is highly expressed in the fly brain, particularly in the *pars intercerebralis* (PI) [18]. Therefore, the PI might be the site for Hmgcr regulation of sleep patterns. To address this, we knocked down *Hmgcr* expression specifically in the PI insulin-producing cells (IPCs) using the Dilp2 (Drosophila insulin-like peptide 2) driver [15]. As published, when crossed to *UAS-GFP*, this line specifically labeled the Dilp2 expressing neurons within the PI (Figure 4a,b). Consistent with pan-neuronal knockdown of *Hmgcr* and fluvastatin-treated flies, we found RNAi-mediated knockdown of *Hmgcr* in Dilp2 neurons led to a significant increase in night sleep duration, while the total sleep duration was normal (Figure 4c–e). The architecture of the sleep parameters was also similar to pan-neuronal *Hmgcr* knockdown or fluvastatin-treated flies during the night phase (Supplementary Figure S3a–g). The increased sleep duration during the night was due to an increase in the mean sleep-episode duration, concomitant with a decrease in the number of sleep episodes, as well as shortening of the sleep latency (Supplementary Figure S3c–f). Unexpectedly, there was also a reduction in locomotor activity while awake (Supplementary Figure S3g). It was noted that sleep latency during the night was also reduced, which did not occur when *Hmgcr* was globally knocked down in the nervous system.

### 2.3. Hmgcr Is Also Required in Pars Intercerebralis DH44 Neurons for Proper Sleep Regulation

**Figure 5:**
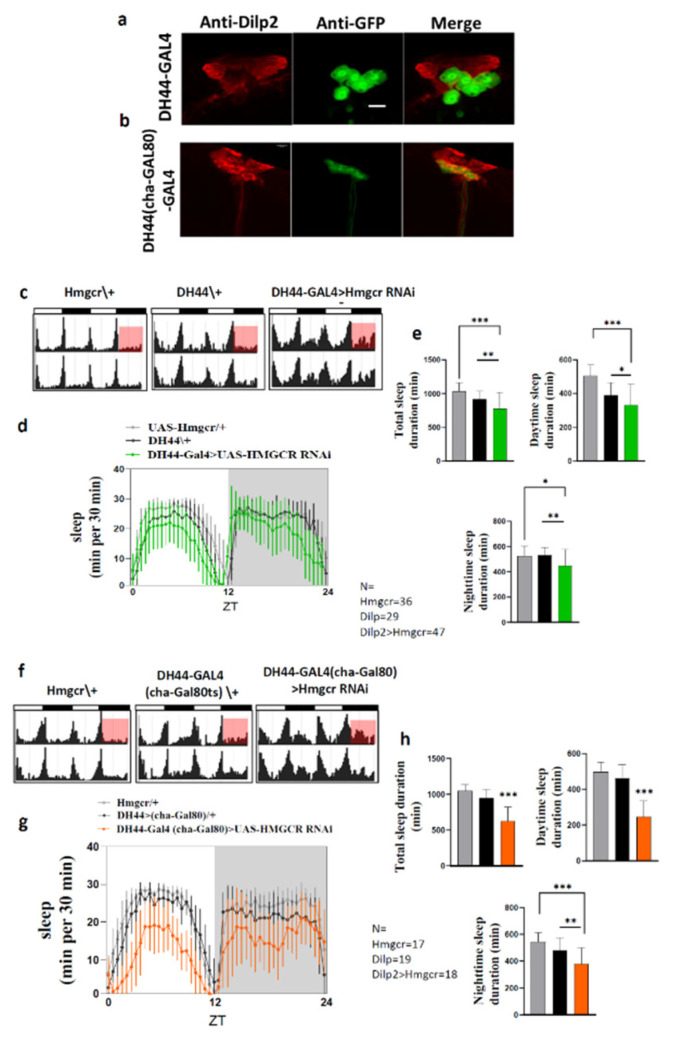
Sleep-activity patterns are affected by Hmgcr knockdown in DH44 neurons. (**a**) *DH44-GAL4* driver crossed to *UAS-GFP*, anti-Dilp2 left, anti-GFP middle and showing complementary to each other. (**b**) *DH44-GAL4* (*tubGAL80^ts^*) driver crossed to *UAS-GFP*, anti-Dilp2 left, anti-GFP middle and showing complementary to each other. Scale bar = 10 μm. (**d**) *DH44-GAL4* (*tubGAL80^ts^*) driver crossed to *UAS-GFP*, anti-Dilp2 left, anti-GFP middle showing complementary to each other. Scale bar = 10 μm. (**c**–**h**) Activity and sleep data for experimental and controls, male flies (*n* = 32) in L:D conditions. (**c**,**f**) White bars indicate daytime (ZT0–12); black bars indicate night (ZT 12–24). The genotypes are indicated above the panels. Red boxes denote the locomotor activity during the night. (**d**,**g**) Architecture of the sleep parameters. Light area indicates daytime (ZT0–12); gray area indicates night (ZT 12–24). The genotypes are indicated above the graph. (**e**,**h**) Graphs indicate locomotor activity, as well as the amount of day and night sleep in minutes. (**e**) Gray bar = w^1118^ > *UAS-Hmgcr* RNAi control, Black bar = *DH44-GAL4* > *w^1118^* control, Green bar = *DH44-GAL4* > *UAS-Hmgcr* RNAi experimental. (**h**) Gray bar = *w^1118^* > *UAS-Hmgcr* RNAi control, Black bar = *DH44-GAL4* (*cha-Gal80^ts^*) > *w^1118^* control, Orange bar = *DH44-GAL4* (*cha-Gal80^ts^*) > *UAS-Hmgcr* RNAi experimental. Activity is measured as the number of beam crossings per minute. Results with error bars are mean ± S.D. To detect the significant difference between groups a Shapiro–Wilk test was performed to determine normality, then a One-way ANOVA and Bonferroni post hoc test was performed to detect significance for total sleep, daytime sleep, and night sleep. * *p* < 0.01, ** *p* < 0.001, *** *p* < 0.0001.

To further identify the function of Hmgcr on sleep parameters within the PI, we used the *DH44-GAL4* driver. DH44 is the Drosophila homolog of Corticotropin-Releasing Factor (CRF) [23]. Previous studies have shown that DH44 expression is restricted to only six neurons within the PI and that these neurons are involved in regulating circadian control of rhythmic locomotor activity [24]. We first mapped the DH44 driver by crossing *DH44-GAL4* flies with *UAS-GFP* flies and verified that the driver expressed in neurons located within the PI and that these neurons were distinguishable from Dilp2 expressing neurons (Figure 5a,b). When we used the *DH44-GAL4* driver to knockdown *Hmgcr* expression, we noticed that the Hmgcr knockdown flies were more active during both the day and night compared to controls. Reducing *Hmgcr* expression specifically in the DH44 neurons also significantly decreased siesta and night sleep levels (Figure 5c–e), mainly due to a reduction in mean sleep-episode duration, as well as an increase in the mean wake-episode duration (Supplementary Figure S4). There was also a decrease in locomotion while awake. To further characterize the effect that loss of *Hmgcr* had in the DH44 neurons, we used another GAL4 driver line, known as *cha-GAL80* [25]. It was designed to specifically target DH44 PI neurons by including the *GAL80* transgene to better define those cells. Using this line demonstrated that the *Hmgcr* RNAi knockdown flies had higher activity levels than controls (Figure 5f). Furthermore, the total sleep duration was significantly reduced during both the day and night, mainly due to prolonged latency of sleep onset at the beginning of the night (Figure 5g,h). Interestingly, these effects were not attributed to an increase in general activity, as locomotion while awake was unchanged between experimental and control groups (Supplementary Figure S5). 

### 2.4. Hmgcr Regulates Sleep through Homeostatic Pathways 

**Figure 6:**
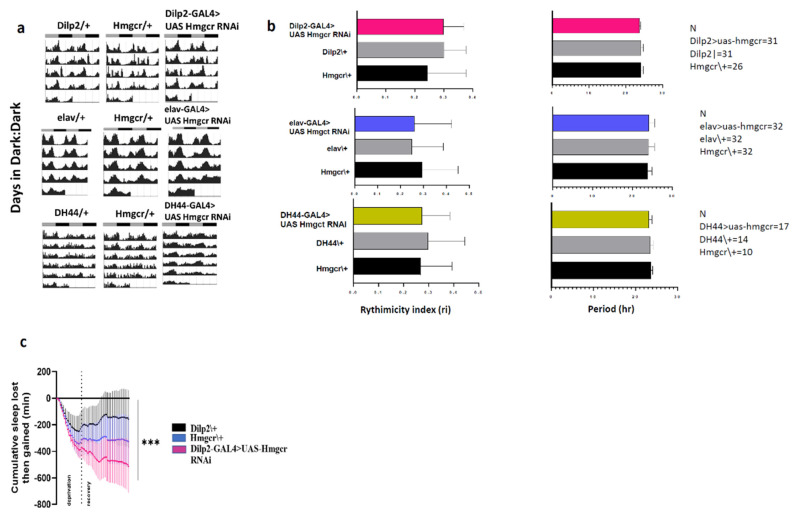
Hmgcr activity in the IPCs is not required for circadian rhythm (**a**) Double plotted actogram for 5 days in dark:dark (DD) constant condition. (**b**) Average rhythmicity index and free-running period. Results with error bars are mean ± S.D. One-way ANOVA was performed, *n* = 30, male flies. (**c**) Sleep deprivation occurred after three baseline days at normal light: dark condition, by switching on the light for 12 h overnight. The regained sleep was measured over the following 24 h. The sleep rebound response was significantly lowered in the Hmgcr knockdown flies (*Dilp2-GAL4* > *UAS-Hmgcr* RNAi) in the PI compared to the controls (*Dilp2-GAL4* > *w^1118^* and *w^1118^* > *UAS-Hmgcr* RNAi). The results with errors bars are mean ± S.D. *n* = 32 for each group, male flies. To detect the significant difference between groups a Shapiro–Wilk test was performed to determine normality, then a one-way ANOVA and Bonferroni post hoc test was performed to detect significant genotype effects. *** *p* < 0.0001.

Generally, sleep is controlled by the interaction between circadian and homeostatic mechanisms. Hmgcr is expressed in PI neurons and previous studies have indicated that the PI, in particular the DH44 neurons [24,26], is implicated in controlling circadian rhythm, as well as being directly connected to clock neurons. In constant darkness, *Hmgcr* knockdown flies displayed identical strength and period of free-running circadian locomotor rhythms as controls flies (Figure 6a,b). To examine if sleep changes in the *Hmgcr* knockdown flies was due to impaired homeostatic regulation, we deprived the flies of sleep for 12 hr overnight. Then, the amount of sleep lost and regained was measured during the subsequent 24 h. Flies, where *Hmgcr* was knocked down specifically within the IPCs (*Dilp2-GAL* > *UAS-Hmgcr* RNAi), lost significantly more sleep than controls (Figure 6c). These data indicate that the homeostatic response to sleep deprivation is affected by the loss of Hmgcr expression in the higher brain center.

## 3. Discussion

Statins are a widely used medication to prolong survival and prevent the consequences of cardiovascular diseases. However, many people reported statins might cause sleep disturbances, including insomnia. Clinical considerations underscore an urgency to identify a firm conclusion on whether statins affect patients’ sleep regulation, as statins discontinuation or non-adherence rates are increasing due to these unsubstantiated side effects [27]. In fact, recent studies found that low adherence to statin therapy was associated with an increased risk of death [28,29]. Statins are among the most effective medications for the prevention of CVDs; therefore, understanding the reasons for low adherence might give clinicians new strategies for increasing patient compliance. Therefore, we performed our study to understand if statins, and their target Hmgcr, can contribute to sleep disturbances.

This is the first study showing that Hmgcr activity is important in the regulation of sleep. Both pan-neuronal *Hmgcr* knockdown and fluvastatin treatment modulate sleep parameters in a similar pattern, suggesting neurons that regulate sleep need Hmgcr activity as part of the sleep circuit. Our results show that Hmgcr is important in the maintenance of sleep because sleep latency and duration of sleep episodes were modulated by inhibiting Hmgcr activity or by knocking down *Hmgcr* expression. Pan-neuronal reduction of *Hmgcr* expression increases sleep amount, mainly due to increased sleep episode duration, decreased frequency number, as well as shortened sleep latency. While this promoting effect on sleep is similar to fluvastatin treatment, fluvastatin exerts more of an effect on sleep latency during the night.

An important anatomical site in the *Drosophila* brain involved in sleep maintenance is the *pars intercerebralis* (PI) [26], and *Hmgcr* is known to be highly expressed in this area. The PI is structurally and developmentally similar to the mammalian hypothalamus [30]. Previous studies found that the PI is responsible for regulating metabolism, sleep, and circadian rhythm, and is recognized as a region for output pathways in *Drosophila* [15]. As in the mammalian hypothalamus, the PI is a heterogeneous region that can be found to have both sleep-promoting and arousal-promoting effects [15,31]. Several genes are reported to be involved in the regulation of sleep in the PI [24,32,33,34]. However, we demonstrate that Hmgcr is crucial for the sleep-wake cycle mediated by the PI, where Hmgcr is among the molecular machinery modulating the shape function of PI neurons in relation to sleep.

Notably, we dissect which neurons within the PI are responsible for the sleep-promoting effects caused by the loss of *Hmgcr* expression. Our results demonstrate that knocking down *Hmgcr* specifically in the insulin-producing Dilp2 neurons induces a similar sleep pattern to pan-neuronal *Hmgcr* knockdown, or when flies are fed fluvastatin. Dilp2 insulin-producing cells (IPCs), located in the PI, are known to be crucial for metabolism homeostasis and sleep regulation [15,35,36]. Specifically, IPCs, which produce insulin-like peptides (e.g., Dilp2) are functionally analogous to pancreatic islet β cells [37,38,39]. Intriguingly, Dilp2 is essential for sleep regulation, perhaps through insulin signaling, and dysregulation of these neural circuits gives rise to disruptions in sleep [40,41]. It is possible that the alteration in sleep due to suppression of *Hmgcr* expression in Dilp2 neurons, or activity caused by fluvastatin treatment, might be linked to disruption of insulin homeostasis, leading to changes in sleep patterns. 

To figure out whether the sleep disruption is due to Hmgcr activity within the PI circadian rhythm linked DH44 neurons, we knocked down *Hmgcr* specifically in DH44 neurons. We found that, in contrast to global Hmgcr suppression, via knockdown or fluvastatin treatment, knocking down *Hmgcr* in only six DH44 PI neurons leads to increased sleep latency and decreased total sleep duration. Interestingly, we also discovered that knocking down *Hmgcr* in DH44 neurons does not change circadian rhythmicity, which suggests that Hmgcr may not be involved in controlling the circadian activity of locomotion. Additionally, we suggest that DH44 neurons could be complementary to Dilp2 neurons. Consistent with this, it has been reported that some of the six DH44 PI neurons co-express Dilp2 in the adult stage, but the functional role of Dilp2 in these neurons is still unclear [42]. The possible role of Hmgcr in Dilp2 neurons, however, might be different from that in DH44 neurons since disruption of Hmgcr in each of these neurons produces an opposite sleep pattern. 

These studies have several strengths, such as we used the genetic *Drosophila* model, together with a pharmacological method, to gain a better understanding of complex behaviors in relation to a statin medication. In addition, we employed different strategies by using various GAL4 driver lines in different subsets of neurons in the same region of the fly’s brain to explore whether Hmgcr can modulate various sleep parameters. The current study also has several limitations; we could not verify whether we successfully knocked down *Hmgcr* in Dilp2 or DH44 neurons in the PI since the number of these cells in the brain are very few, and thus changes in *Hmgcr* RNA expression could not be detected easily in quantitative real-time PCR. However, using a global neuronal *Hmgcr* knockdown (*elav-GAL4*), we successfully demonstrate that *Hmgcr* transcripts were significantly lower than controls in *Hmgcr* knockdown flies (Supplementary Figure S6). Moreover, the study did not cover the sleep changing effects of Hmgcr disruption specifically in peripheral neurons, which would probably strengthen our findings that the fluvastatin effect or Hmgcr disruption on sleep structure are mainly due to Hmgcr’s role in neurons found in the *pars intercerebralis*. Furthermore, we did not rescue the fluvastatin/Hmgcr disruption-induced sleep phenotype, which would help us to understand the underlying mechanisms.

Overall, we show that Hmgcr is important for sleep regulation in flies and that changes in its activity in the fly brain could cause a discrete effect on sleep structure. This study suggests that statin-associated sleep problems might be attributed to the inhibition of Hmgcr in specific PI neurons within the fly brain. Generally, statins affect sleep architecture and increase sleep consolidation due to a reduced number of sleep episodes and increased length of each episode, as well as decreasing sleep latency in the flies. Further studies are needed to discover if these findings can translate to mammals. This study demonstrates that the fly model is very useful for understanding the effects of statins on the molecular and anatomical regulation of sleep.

## 4. Materials and Methods

### 4.1. Fly Stocks and Maintenance

Flies were reared on standard fly food (Jazz mix, Fisher Scientific, Gothenburg, Sweden) and supplemented with yeast extract (VWR, Stockholm, Sweden). Flies were maintained at 25 °C (unless otherwise stated for specific experiments) in an incubator at 60% humidity on 12:12 h light:dark cycle. All flies were crossed onto the same *w^1118^* background. The following strains were used w[*]; P{w[+mC]=GAL4-elav.L}3 (#8760), w[*]; P{w[+mC]=Ilp2GAL4.R}2/CyO(#37516),y[1]v[1];P{y[+t7.7]v[+t1.8]=TRiP.HMC03053}attP40 (#50652), w[1118]; P{y[+t7.7] w[+mC]=GMR65C11-GAL4}attP2 (#39347), Canton S and *OregonR-C, w^1118^* were received from the Bloomington Stock Center (Bloomington, Indiana, IN, USA). The second *UAS-Hmgcr* RNAi KK line from Vienna Drosophila Resource Center (Vienna, Austria) (#101807). The *w[1118]; P{w[+mC]=DH44-Gal4}vie72a/Cyo; P{w[+mC]=Cha-Gal80}/TM3,Ser* flies were a generous gift from Dr. Young-Joon Kim (Gwangju Institute of Science and Technology) [25]. In addition, the *elav-Gal80* transgene line was generously gifted from Dr.Yuh Nung Jan and Dr. Suaun Younger (University of California, San Francisco, CA, USA).

### 4.2. Locomotor Activity Assay

Activity, sleep, and circadian rhythm was measured based on the assessment of locomotor activity using the Drosophila Activity Monitor System (DAMS) (TriKinetics Inc., Waltham, MA, USA) for 5 days. All experiments were performed on an individual, adult flies (aged 3–5 days) at 25 °C, 60% humidity and under 12:12 light:dark conditions. The flies were loaded into the DAMS tubes. During the measurements, the flies’ activity was registered continuously via infrared beams, and data from activity channels was uploaded to a PC and subsequently analyzed in Matlab via a specific analysis software (SCAMP, TriKinetics Inc., Waltham, MA, USA) [43]. Furthermore, sleep is calculated as no movement for five minutes [44]. Sleep latency is the time from light off to the first sleep episode.

### 4.3. Statin Experiments

For experiments involving statin drugs, we used fluvastatin (#SML0038, SigmaAldrich, Stockholm, Sweden), which was solubilized in water and mixed into the regular lab fly food, at concentrations of 0, 0.05, 0.5, or 1 mM. Then the DAMS was run to monitor sleep-activity patterns at 12:12 LD condition at 25 °C for five days. Statin experiments were performed using male, wild-type flies of 3–5 days of age.

### 4.4. Immunostaining

Brains from adult flies aged 5–7 days were dissected in phosphate-buffered saline (PBS) and then fixed in 4% ice-cold paraformaldehyde (PFA) for 4 h, before being rinsed into PBS for 1 h. Samples were then incubated for 48 h at 4°C in primary antibodies diluted in PBS with 0.5% Triton X-100 (PBST). After washes in PBST for 1 h at room temperature, the samples were incubated with secondary antiserum for 48 h at 4°C. Finally, all samples were washed with PBST and then maintained with 80% glycerol. The following primary antisera were used: rabbit anti-Dilp2 at dilution of 1:2000 (both were kindly donated by J. A. Veenstra, Bordeaux, France). mouse anti-GFP at a dilution of 1:1000. For detection of primary antisera, we used Alexa 546 tagged goat anti-rabbit antiserum and Alexa 488 tagged goat anti-mouse antiserum (Invitrogen, Stockholm, Sweden) at a dilution of 1:1000. After washes, tissues were mounted in 80% glycerol in PBS. Tissue samples were imaged with a Zeiss LSM 710 META confocal microscope (Jena, Germany) using 20× or 63× oil immersion objectives. Confocal images were processed with FIJI immunofluorescence levels were recalculated to correct total cell fluorescence (CTCF) using FIJI software.

### 4.5. Statistical Analysis

All statistical analyses were performed in GraphPad Prism 8. Data were tested for normality using the Kolmogorov–Smirnov test. Normally distributed data were further analyzed with one-way analysis of variance (ANOVA) followed by Tukey’s test post hoc test. Data that were not normally distributed were analyzed using the Kruskal–Wallis test. A P-value was lower than 0.05 was considered as a statistical indicator for significant differences between the groups.

## 5. Conclusions

Our study shows that Hmgcr is involved in regulating sleep structure in Drosophila. By using various GAL4 driver lines we determined that Hmgcr can modulate sleep parameters in both the insulin-producing neurons (Dilp2-GAL4) as well as neurons that express the corticotropin-releasing hormone (CRH) homolog DH44 (DH44-GAL4). Furthermore, in flies, fluvastatin affects sleep architecture by increasing sleep consolidation, due to a reduced number of sleep episodes, with a concurrent increase in episode length. Fluvastatin also as well as decreases sleep latency in the flies. In conclusion, this study shows that inhibition of Hmgcr in specific PI neurons within the fly brain is sufficient to disrupt normal sleep patterns.

## Data Availability

Data is contained within the article and supplementary material.

## Supplementary Materials

The following are available online at https://www.mdpi.com/article/10.3390/ph15010079/s1, Figure S1: Hmgcr RNAi in the whole neurons reduces the generalised activity at Light: Dark condition, Figure S2: The average activity of the flies-treated statins decreased in a dose-dependent manner at Light: Dark condition, Figure S3: Sleep-activity patterns are affected by Hmgcr knockdown in the Dilp2 neurons, Figure S4: Sleep parameters are modulated by Hmgcr knockdown in the DH44 neurons, Figure S5: Sleep parameters are affected by Hmgcr knockdown in the DH44 neurons, Figure S6: Quantification of Hmgcr RNAi line efficiency.
