# Supplementary material for: The Statin Target HMG-Coenzyme a Reductase (Hmgcr) Regulates Sleep Homeostasis in Drosophila
Source: Pharmaceuticals (Basel). 2022 Jan 10;15(1):79. doi: 10.3390/ph15010079 (PMC8781969; doi:10.3390/ph15010079)
Supplement: Supplementary file 1 [file pharmaceuticals-15-00079-s001.zip › pharmaceuticals-1447121-supplementary.pdf]

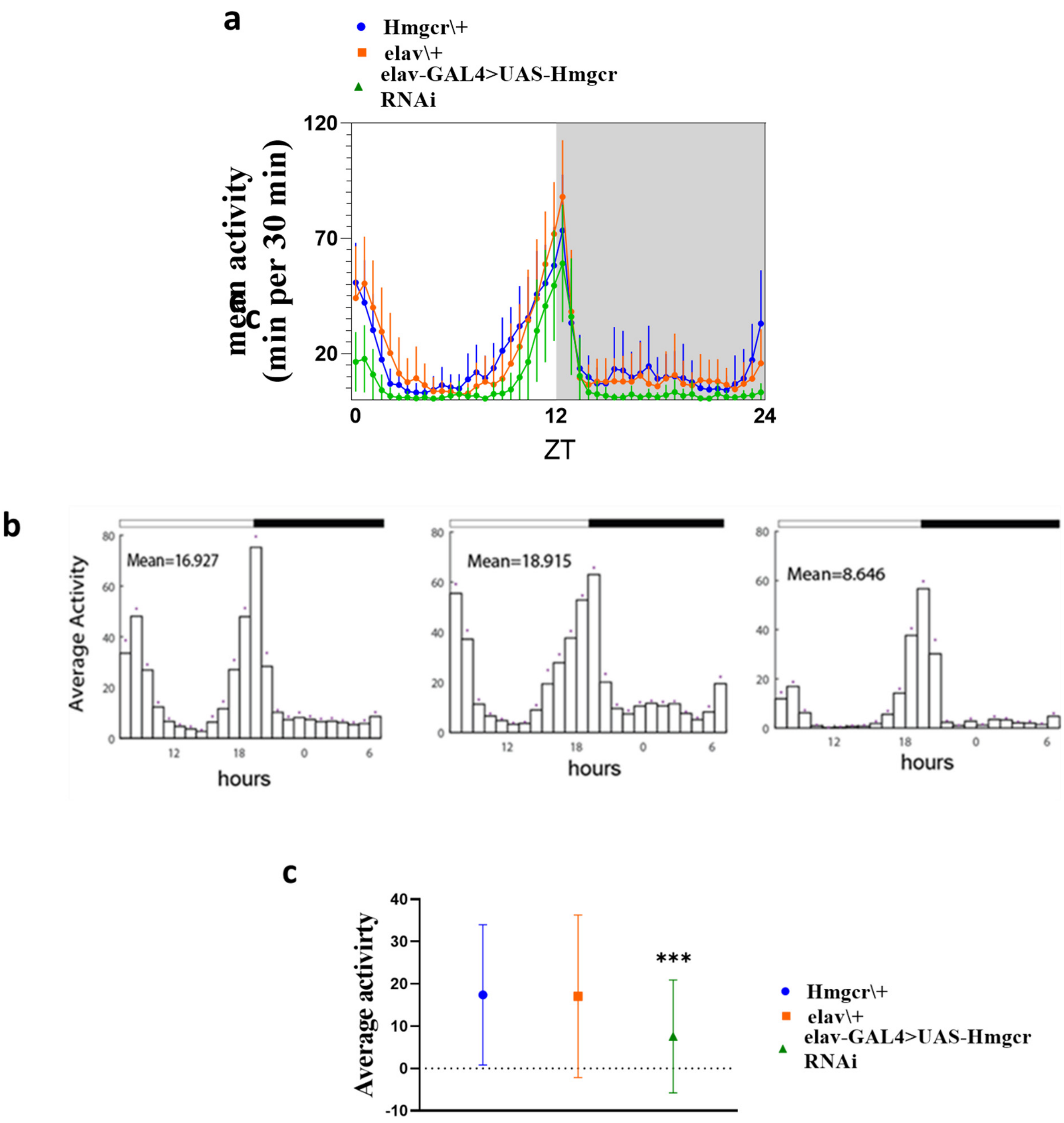

Figure S1. Hmgcr RNAi in the whole neurons reduces the generalised activity at Light: Dark condition. (**a-c**) The average activity per 30 min was recorded for control (elav/+ and Hmgcr/+) and experimental (elav-GAL4>UAS-Hmgcr RNAi) male flies. White bars denote daytime (ZT0–12); black bars indicate night (ZT 12–24). All results represented with errors bars are mean ± S.D. n=32 for each group. One-way ANOVA was performed to detect significant genotype effects for average activity. *** P< 0.0001.

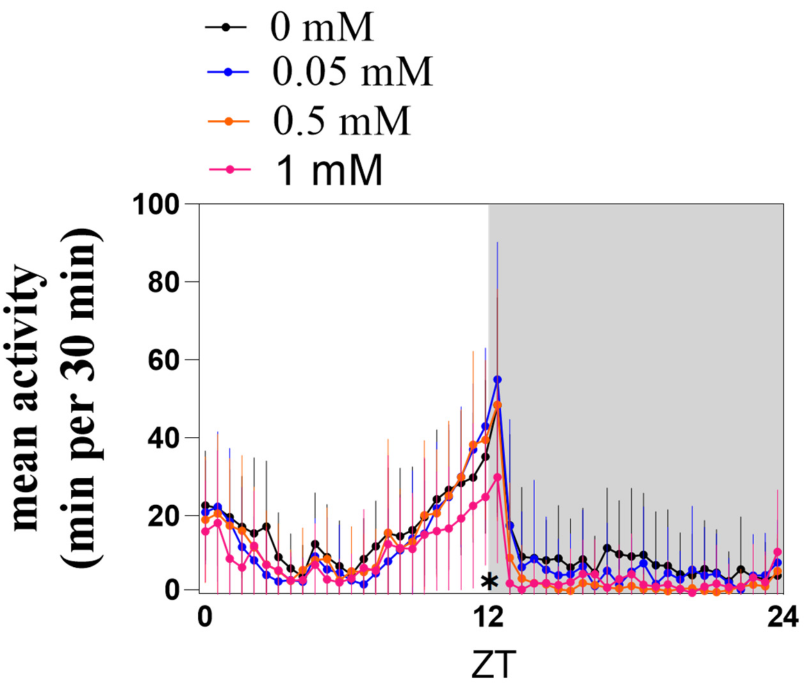

Figure S2. The average activity of the flies-treated statins decreased in a dose-dependent manner at Light: Dark condition. The activity plot shows the average activity per 30 min in flies treated with different concentration of fluvastatin medication. One-way ANOVA was performed to detect significance for average activity. * P< 0.01.

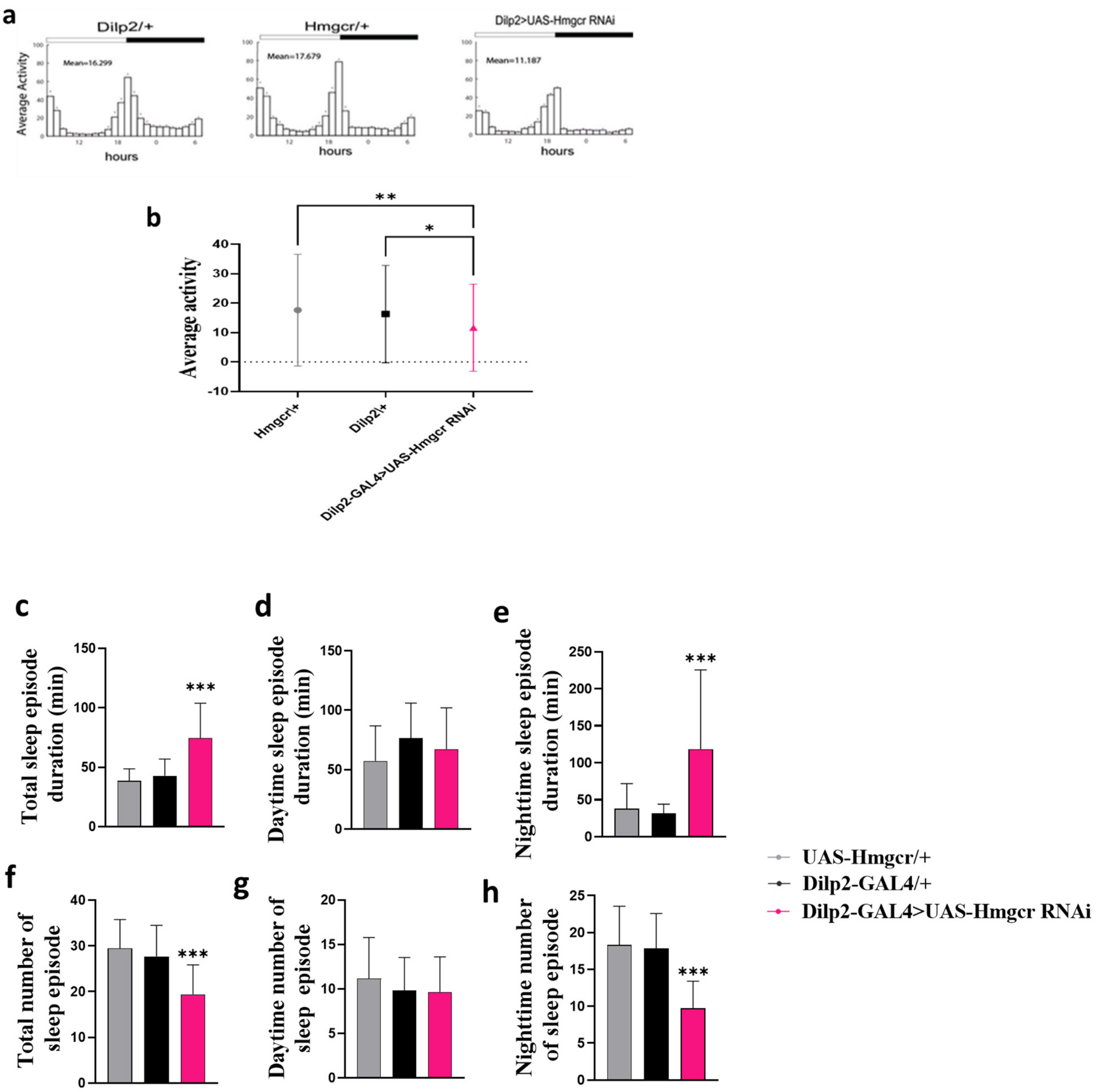
a
Dilp2/+
Hmgcr/+
Dilp2>UAS-Hmgcr RNAi
Average Activity
Mean=16.299
Mean=17.679
Mean=11.187
hours
b
Average activity
Hmgcr\+
Dilp2\+
Dilp2-GAL4>UAS-Hmgcr RNAi
c
Total sleep episode duration (min)
d
Daytime sleep episode duration (min)
e
Nighttime sleep episode duration (min)
f
Total number of sleep episode
g
Daytime number of sleep episode
h
Nighttime number of sleep episode
UAS-Hmgcr/+
Dilp2-GAL4/+
Dilp2-GAL4>UAS-Hmgcr RNAi

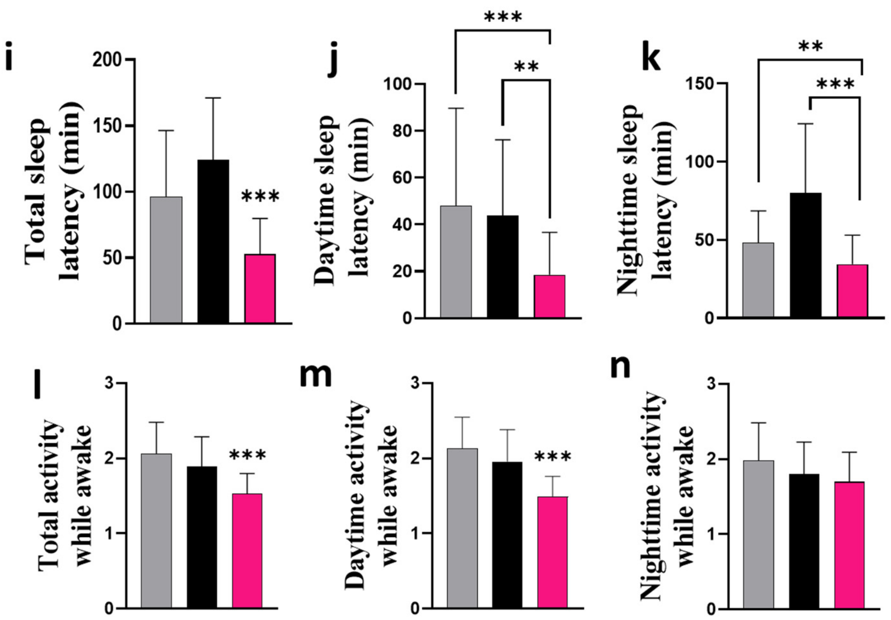

Figure S3. Sleep-activity patterns are affected by Hmgcr knockdown in the Dilp2 neurons. (**a** and **b**) The average activity was recorded for control (Dilp2-GAL4/+ and UAS-Hmgcr RNAi/+) and experimental (Dilp2>UAS-Hmgcr RNAi) male flies. The white bars denote daytime (ZT0–12); black bars indicate night (ZT 12–24). (**c-n**) mean sleep-episode duration, sleep-episode frequency, sleep latency, wake-episode duration and activity while awake of the control groups and experimental group. The results with errors bars are mean ± S.D. n=32 for each group. One-way ANOVA was performed to detect significant genotype effects. * P< 0.01, ** P<0.001, *** P<0.0001.

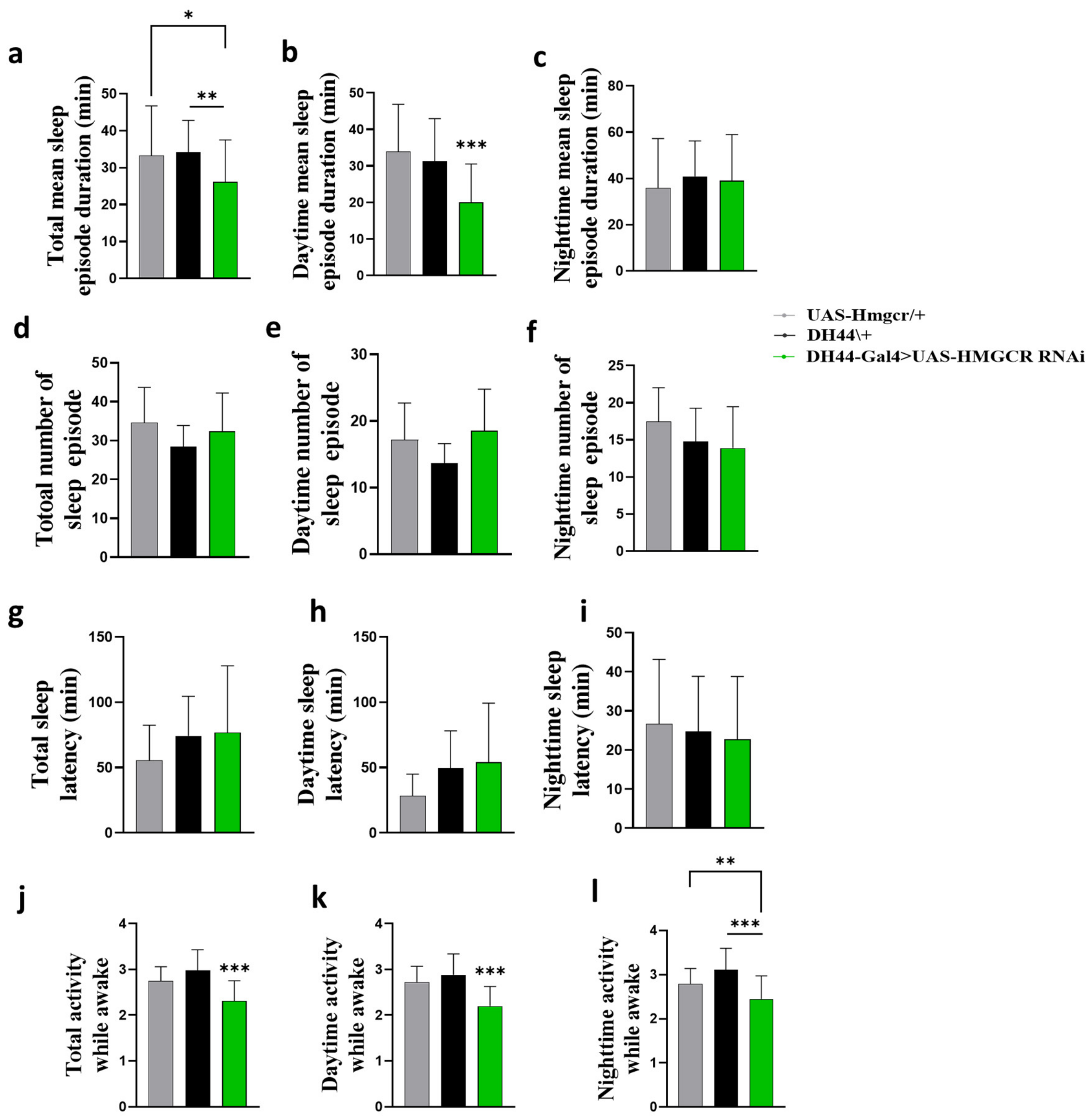

Figure S4. Sleep parameters are modulated by Hmgcr knockdown in the DH44 neurons. (**a-l**) The sleep parameters (sleep latency, mean sleep episode duration, number of the sleep episode, mean wake episode duration and activity while awake) for control (DH44-GAL4/+, UAS-HMGCR RNAi/+) and experimental groups (DH44-GAL4>UAS-HMGCR RNAi). The results with errors bars are mean ± S.D. n=32 for each group, male flies. One-way ANOVA was performed to detect significant genotype effects. P-values= *<0.01, **<0.001, ***<0.0001.

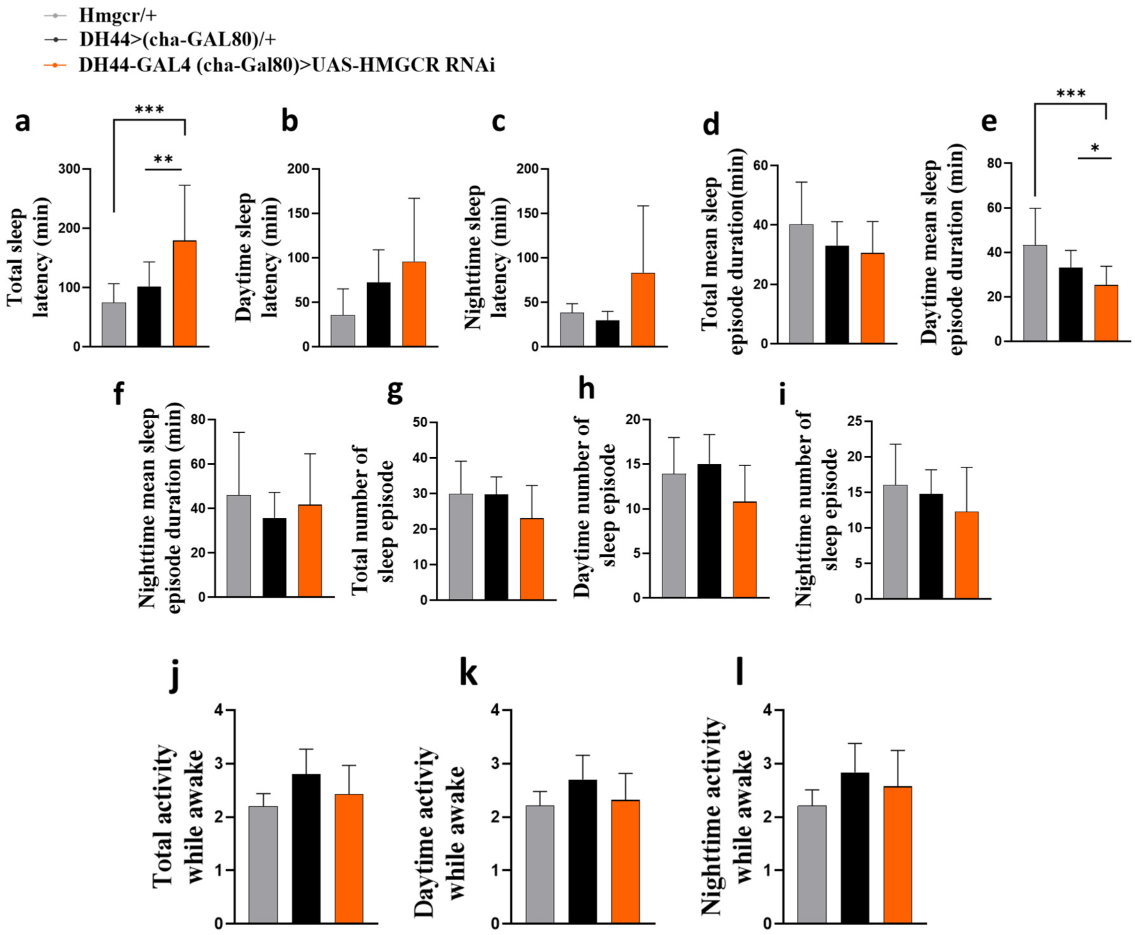

Figure S5. Sleep parameters are affected by Hmgcr knockdown in the DH44 neurons. (**a-l**) Sleep parameters are modulated by Hmgcr knockdown in the DH44 neurons. The sleep parameters (sleep latency, mean sleep episode duration, number of the sleep episode, mean wake episode duration and activity while awake) for control (DH44-GAL4(cha-GAL80)/+, UAS-HMGCR RNAi/+) and experimental groups (DH44-GAL4(cha-GAL80)>UAS-HMGCR RNAi). The results with errors bars are mean ± S.D. n=32 for each group, male flies. One-way ANOVA was performed to detect significant genotype effects. P-values= *<0.01, **<0.001, ***<0.0001.

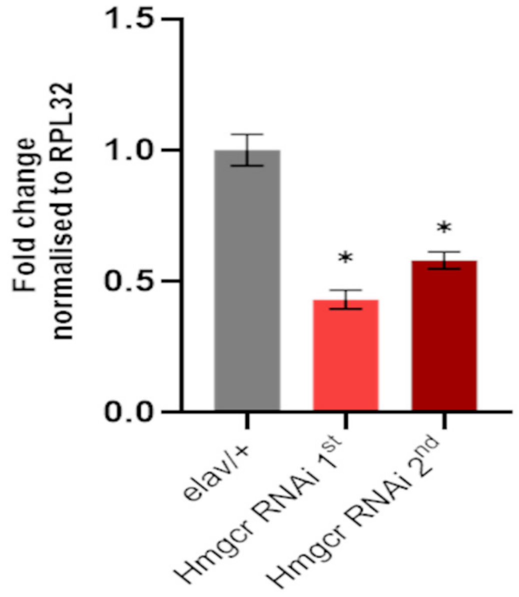

Figure S6. Quantification of Hmgcr RNAi line efficiency. The total mRNA was extracted from the whole fly; then, the RT-qPCR was performed. n=7, male flies, error bars represent ±S.D., * $P < 0.05$. Forward primer: GCTGCACTGCCGTACTGTA; reverse primer: AATGCCCAGCACATATTTGGA.
